# Engineering a HEK-293T exosome-based delivery platform for efficient tumor-targeting chemotherapy/internal irradiation combination therapy

**DOI:** 10.1186/s12951-022-01462-1

**Published:** 2022-05-31

**Authors:** Congcong Wang, Ning Li, Yutian Li, Shasha Hou, Wenxin Zhang, Zhaowei Meng, Shen Wang, Qiang Jia, Jian Tan, Renfei Wang, Ruiguo Zhang

**Affiliations:** 1 Department of Nuclear Medicine, Tianjin Medical University General Hospital, No. 154 Anshan Road, Heping District, Tianjin, 300052 China; 2 Department of Nuclear Medicine, The Affiliated Hospital of Qingdao University, No. 16 Jiangsu Road, Shinan District, Qingdao, 266003 Shandong China; 3 Department of Radiology, Qingdao Women and Children’s Hospital, No. 217 Liaoyang West Road, Shibei District, Qingdao, 266000 Shandong China; 4 Department of Nuclear Medicine, Shanghai Tenth People’s Hospital, Tongji University School of Medicine, Shanghai, 200072 China

**Keywords:** Exosome, iRGD peptide, Radioiodine-131, Anaplastic thyroid carcinoma, Tumor targeting, Combination therapy

## Abstract

Exosomes are nanoscale monolayer membrane vesicles that are actively endogenously secreted by mammalian cells. Currently, multifunctional exosomes with tumor-targeted imaging and therapeutic potential have aroused widespread interest in cancer research. Herein, we developed a multifunctional HEK-293T exosome-based targeted delivery platform by engineering HEK-293T cells to express a well-characterized exosomal membrane protein (Lamp2b) fused to the αv integrin-specific iRGD peptide and tyrosine fragments. This platform was loaded with doxorubicin (Dox) and labeled with radioiodine-131 (^131^I) using the chloramine-T method. iRGD exosomes showed highly efficient targeting and Dox delivery to integrin αvβ3-positive anaplastic thyroid carcinoma (ATC) cells as demonstrated by confocal imaging and flow cytometry in vitro and an excellent tumor-targeting capacity confirmed by single-photon emission computed tomography-computed tomography after labeling with ^131^I in vivo. In addition, intravenous injection of this vehicle delivered Dox and ^131^I specifically to tumor tissues, leading to significant tumor growth inhibition in an 8505C xenograft mouse model, while showing biosafety and no side effects. These as-developed multifunctional exosomes (denoted as Dox@iRGD-Exos-^131^I) provide novel insight into the current treatment of ATC and hold great potential for improving therapeutic efficacy against a wide range of integrin αvβ3-overexpressing tumors.

**Graphical Abstract:**

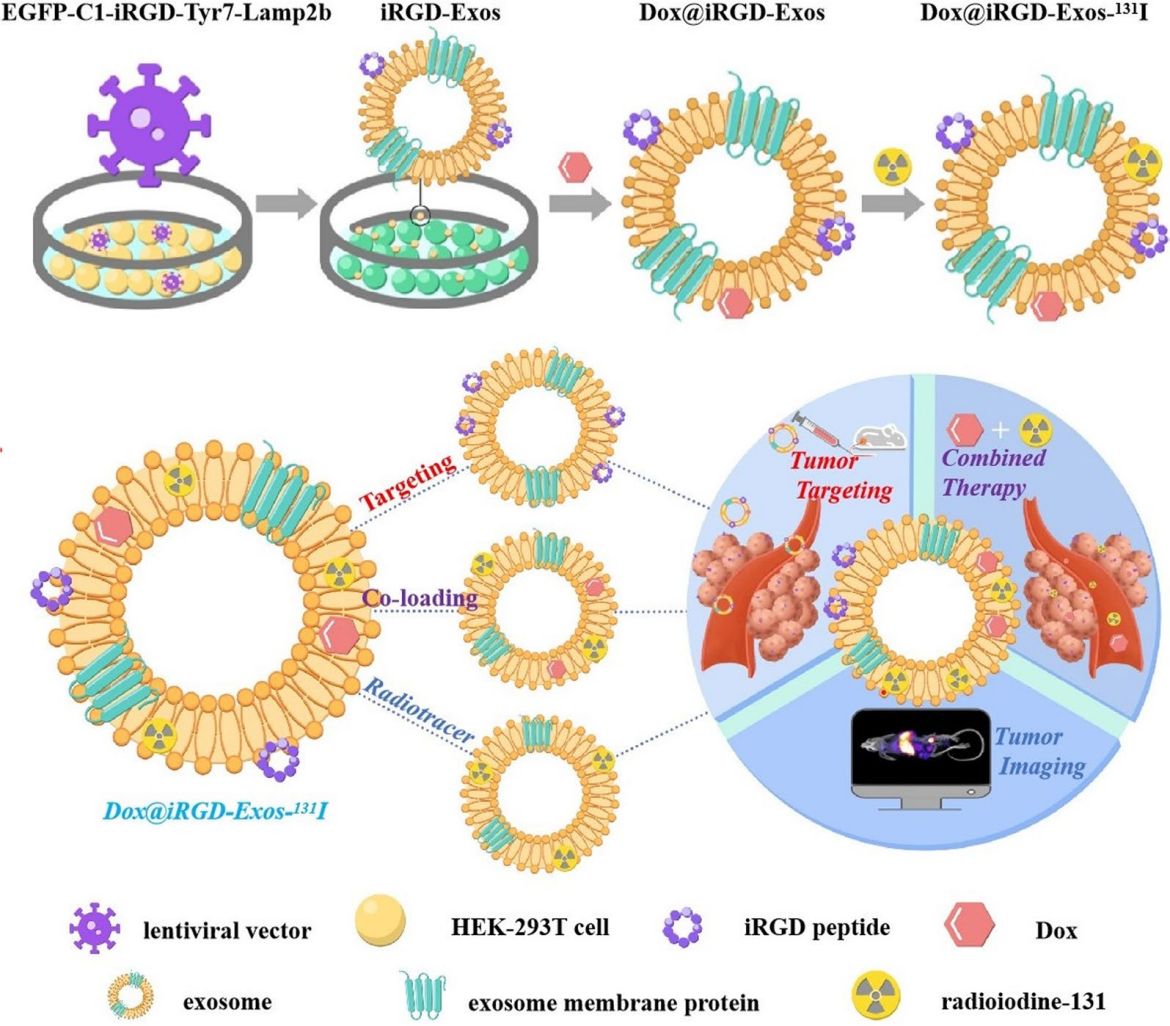

**Supplementary Information:**

The online version contains supplementary material available at 10.1186/s12951-022-01462-1.

## Introduction

Anaplastic thyroid carcinoma (ATC) is a rare, undifferentiated, highly lethal form of cancer, and diagnosed patients usually have a rapidly growing neck mass, neck pain, vocal cord paralysis, dysphagia, and dyspnea [1–3]. The median survival time after diagnosis is approximately 5–12 months, and the 1-year overall survival rate is less than 20% [2, 4].

Radioiodine-131 (^131^I), as a postoperative adjuvant therapy, has been extensively used for diagnosis and therapy of differentiated thyroid cancer (DTC) metastasis for many years [5, 6]. However, patients with ATC cannot benefit from ^131^I therapy due to a lack of sodium iodide transporter expression, structure, or transport [2, 7]. For many decades, chemotherapy has been the cornerstone of cancer therapy because it can reduce recurrence or metastasis and prolong the overall survival of patients with cancer [8, 9]. Doxorubicin (Dox) is a anthracycline therapeutic agent that can intercalate into double-stranded DNA to prevent DNA replication and RNA transcription by inhibiting DNA and RNA polymerase; therefore, it has been widely used to treat breast cancer, ovarian cancer, leukemia, and other malignant tumors [8, 10–14]. Moreover, Dox is the only chemotherapy drug approved by the U.S. Food and Drug Administration (FDA) for the treatment of metastatic thyroid carcinoma, as it shows significantly better efficacy in lung metastases than in lymph node or bone metastases [15, 16]. However, the dense extracellular matrix and abnormal vascular structure in tumor tissues constitute a complex microenvironment, causing most chemotherapeutic drugs to be distributed only around the tumor vasculature rather than accumulating in the tumor parenchyma [17]. Notably, a low drug concentration in the tumor parenchyma is the main cause of tumor chemoresistance and remains one of the primary obstacles in cancer therapy [9, 18, 19].

Many attempts have been made to solve the above problems by designing multidrug nanoscale delivery systems, such as those incorporating nanosized transition metals, liposomes, polymers, and exosomes [20–22]. Photothermal therapy using nanoparticles to transduce near-infrared laser radiation into local heat to kill tumor cells has the advantages of minimal invasiveness, high efficiency, few adverse reactions and effective tumor metastasis inhibition [23, 24]. Exosomes, as nanoscale membrane particles secreted by cells, can carry a substantial number of drugs and achieve efficient cancer theranostics through surface modification [25, 26]. In recent years, engineered exosomes have been used as a new generation of codelivery vehicles and produced enticing results to reverse tumor drug resistance and enhance the effects of tumor-targeted therapy [27–29]. Integrin αvβ3, an important member of the integrin family, has been widely studied due to its crucial role in tumor angiogenesis. It has been confirmed that integrin αvβ3 is significantly underexpressed or not expressed on the surface of normal tissue cells but is significantly overexpressed in tumor cells and tumor neovascular endothelial cells [30–32]. The iRGD peptide can inherently bind with integrin αvβ3 on the tumor vascular endothelium and on tumor cells [33, 34]. Thus, it is reasonable to envisage the application of exosomes modified with iRGD as a new nanoplatform for tumor angiogenesis imaging and targeted therapy.

**Scheme 1:**
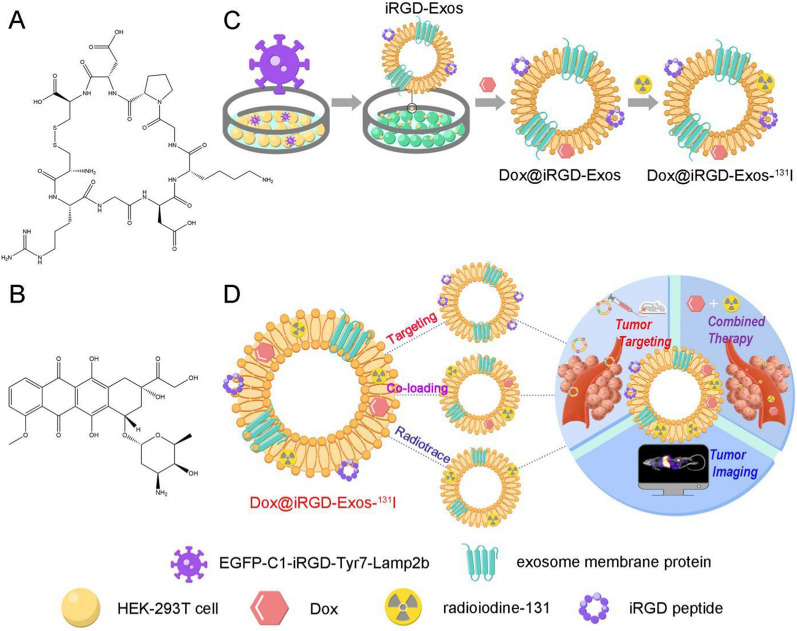
**A** Chemical structure of the iRGD peptide. **B** Chemical structure of Dox. **C** Schematic illustration of the procedure to produce engineered HEK-293T exosomes (donated as Dox@iRGD-Exos-^131^I). **D** Schematic representation of Dox@iRGD-Exos-^131^I for efficient chemotherapy combined with ^131^I labeling. Making full use of lentiviral vector technology and the structure of the exosomal membrane, we developed an engineered HEK-293T exosome-based delivery system that intelligently integrates three functions: tumor targeting, ^131^I and Dox coloading with high payloads, and tumor-targeted therapy. After intravenous injection, Dox@iRGD-Exos-^131^I efficiently accumulated at the tumor site, resulting in a significantly enhanced antitumor chemo/internal irradiation combination therapy effect

Motivated by this rationale, we developed a novel concept by exploring the feasibility of using HEK-293T exosomes as a nanoscale codelivery vehicle through the integration of three outstanding functions: tumor targeting, efficient and flexible coloading of Dox and ^131^I, and tumor imaging and therapy. Specifically, as shown in Scheme 1, to achieve the above three functions, we made full use of lentiviral vector technology and the structure and biochemical composition of the exosomal membrane. (i) First, we constructed an EGFP-C1-iRGD-Tyr7-Lamp2b lentiviral vector containing exosomal membrane protein (Lamp2b) genes, tyrosine genes, and iRGD peptide genes for transfection into HEK-293T cells to obtain a new type of exosome (iRGD-Exos). The surface of these exosomes was enriched with multiple tyrosine fragments, iRGD peptides and Lamp2b. (ii) Subsequently, Dox was loaded into the exosomal phospholipid bilayer membrane structure and ^131^I was labeled onto the tyrosine-rich sites of the exosomal membrane surface using the chloramine-T method. Based on this strategy, in the present study, we developed a HEK-293T exosome-based multifunctional delivery platform (denoted as Dox@iRGD-Exos-^131^I) and observed its targeting ability and tumor suppressive effects on ATC through a series of in vivo and in vitro experiments. Thus, we provide novel insight into the current ATC treatment and explore the potential for improving therapeutic efficacy against a wide range of integrin αvβ3-overexpressing tumors.

## Materials and methods

### Human tissues and ethics statements

Paraffin-embedded tissue samples of ATC cancerous tissues and paired adjacent noncancerous tissues were collected from 8 ATC patients (6 women and 2 men, age range: 14–62 years) in the Department of Pathology of our hospital from 2005 to 2020. The study protocol was approved by the Ethics Committee of Tianjin Medical University General Hospital and conformed to the standards set by the Declaration of Helsinki. All patients who participated in this study provided written informed consent.

### Immunohistochemistry

Paraffin-embedded tissue samples (including ATC cancerous tissues and the paired adjacent noncancerous tissues) from 8 ATC patients (8 pairs, 16 samples total) were sectioned and stained with a 1:200 dilution of anti-integrin αvβ3 polyclonal antibody (Bioss, China) according to the manufacturer’s protocol. Positive staining was identified with a DAB system (Jinqiao, Zhongshan, China). Six regions were randomly selected for each specimen.

### Cell line and culture

The human embryonic kidney epithelial cell line HEK-293T, the human ATC cell lines Hth7, 8505C, THJ16T, and Cal-62 and the human normal thyroid cell line Nthy-ori 3-1 were purchased from the Shanghai Institute of Cell Biology of Chinese Academy of Sciences (Shanghai, China). HEK-293T cells and Hth7 cells were propagated in Dulbecco’s modified Eagle’s medium (DMEM, Gibco, USA) supplemented with 10% fetal bovine serum (FBS, Gibco, USA) and 1% penicillin/streptomycin (Solarbio, China). 8505C, THJ16T and Cal-62 cells were cultured in RPMI-1640 medium (Gibco, USA) supplemented with 10% FBS and antibiotics. Nthy-ori 3-1 cells were cultured in F12K medium (Gibco, USA) supplemented with 10% FBS and antibiotics. All cells were cultured in culture dishes and maintained in 5% CO_2_ at 37 °C.

### Protein extraction and western blotting

The expression of integrin αvβ3 was evaluated by western blot and normalized to that of β-actin [34]. Hth7, 8505C, THJ16T, Cal-62, and Nthy-ori 3-1 cells were plated in 6-well plates and cultured in cell-based medium containing 10% FBS for 24 h. Cells at ~ 80% confluence were used for subsequent protein extraction. The different cells were washed with 1 × PBS 3 times and then lysed in RIPA buffer supplemented with PMSF for 15 min at 4 ℃. Proteins were separated by 10% SDS-PAGE and then transferred to PVDF membranes (Millipore, USA). The immunoblots were blocked with 1 × PBS-5% fat-free dried milk for 1.5 h at room temperature and then incubated at 4 ℃ with anti-integrin αvβ3 polyclonal antibody (1:1000, Bioss, China) and anti-β-actin antibody (1:5000, Abcam, UK) for more than 16 h. After incubation with the HRP-conjugated secondary antibody (1:5000, Bioss, China) for 1 h, the PVDF membranes were visualized with an enhanced chemiluminescence system kit (Millipore, Bedford, MA, USA), and the grayscale of the strip was analyzed by ImageJ [28, 34, 35].

### Construction of the EGFP-C1-iRGD-Tyr7-Lamp2b lentiviral vector and subsequent infection

The EGFP-C1-iRGD-Tyr7-Lamp2b lentiviral vector and the negative control vector EGFP-C1-blank-Tyr7-Lamp2b were purchased from Shanghai Jikai Gene Chemical Technology Co., Ltd (GV367 vector, AgeI/NheI digestion), and cocultured with HEK-293T cells at 37 ℃ for 48 h, respectively. Then, stable iRGD/blank-Tyr7-EGFP-293T cells were screened in complete DMEM containing 2 μg/mL puromycin (Solarbio, China).

### Isolation of iRGD-Exos from the medium

After culturing the stable iRGD-Tyr7-EGFP-293T cells for 48 h, the cell culture supernatant was collected, and the iRGD-Exos were isolated by differential centrifugation according to the related literature [20, 28, 36]. All procedures were carried out at 4 ℃. First, the cell culture supernatant containing iRGD-Exos was centrifuged at 3000×*g* for 30 min to remove dead cells and other debris. Then, the supernatant was centrifuged at 10,000×*g* for 45 min to remove larger-sized vesicles. Finally, the supernatant was filtered through a 0.2-mm filter and centrifuged at 100,000×*g* for 90 min, and iRGD-Exos were collected from the sediment and resuspended in 100 μL of 1 × PBS. The protein concentration of iRGD-Exos was measured with a BCA protein determination assay according to the manufacturer’s protocol and recorded.

### Preparation and characterization of Dox@iRGD-Exos-^131^I

To construct Dox@iRGD-Exos-^131^I, iRGD-Exos were first isolated by differential centrifugation. Then, 200 μL of iRGD-Exos solution (1 mg/mL) and 40 μL of Dox (2 mg/mL) were moderately stirred for 2 h at 4 ℃. Subsequently, following a previously reported procedure, we labeled Dox@iRGD-Exos with ^131^I using the chloramine-T method [37, 38]. A total of 740 MBq of Na^131^I and 100 μL chloramine-T solution (5 mg/mL) were added to the Dox@iRGD-Exos solution. After 120 s of shaking and incubation, 100 μL of sodium metabisulfite solution (5 mg/mL) was added to terminate the oxidation reaction. Finally, the product of Dox@iRGD-Exos-^131^I was separated by several centrifugations (100,000×*g*, 90 min, 4 ℃), and resuspended in 500 μL of 1 × PBS. The labeling efficiency and radiochemical purity were determined by instant thin-layer chromatography (TLC) with an AR-2000 radio-TLC imaging scanner (Bioscan, Poway, CA, USA) [39, 40].

The zeta potentials, sizes and polydispersity indices (PDIs) were determined for the different samples using dynamic light scattering (DLS; Zetasizer Nano ZS90, Malvern, UK). Morphology and size were observed by high-resolution transmission electron microscopy (TEM; HT7700, Hitachi, Japan) at 80 kV. The size distribution and particle concentration were analyzed and recorded with the NanoSight NS300 system (Malvern, UK) and Nanoparticle Tracking Analysis software (NTA, version 2.3).

The exosomal markers Alix, TSG101, and CD9 were confirmed by western blotting analysis. In brief, blank-Tyr7-EGFP-293T cells, iRGD-Tyr7-EGFP-293T cells, blank-Exos, and iRGD-Exos were lysed with RIPA buffer supplemented with PMSF, separated via SDS-PAGE, transferred to PVDF membranes, and blocked with 1 × PBS-5% fat-free dried milk as described by the manufacturer. Then, the PVDF membranes were incubated at 4 °C overnight with anti-Alix (1:1000, Santa Cruz, USA), anti-TSG101 (1:200, Santa Cruz, USA), and anti-CD9 (1:1000, Abcam, UK) antibodies. After incubation with HRP-conjugated secondary antibody, the PVDF membranes were visualized with a gel imaging system (Millipore, Bedford, MA, USA).

### Quantitation of Dox loaded into the exosomes

When 80 μg Dox and 740 MBq Na^131^I were added to 200 μg of iRGD-Exos, Dox@iRGD-Exos-^131^I were obtained via centrifugation (100,000×*g*, 90 min, 4 ℃). After washing several times with 1 × PBS, the supernatants were collected, and Dox@iRGD-Exos-^131^I were resuspended in 500 μL of 1 × PBS. The amount of free Dox in the supernatants was measured and calculated from the standard calibration curve based on the absorbance at λ = 485 nm measured by using an ultraviolet–visible (UV–Vis) spectrophotometer (UV-3600 plus, Hitachi, Japan). The loading efficiency (%) of Dox was calculated as follows [20, 41]:

1 $$A=\frac{(\mathrm{B}-\mathrm{C})100\mathrm{\%}}{B}.$$

where A is the loading efficiency (%) of Dox; B is the original weight of Dox; and C is the weight of Dox in the supernatants.

### Stability of Dox@iRGD-Exos-^131^I

Purified blank-Exos and Dox@iRGD-Exos-^131^I were transferred to glass vials and incubated in 1 × PBS at 4 °C and serum at 37 °C, respectively. Then, blank-Exos and Dox@iRGD-Exos-^131^I were separated by centrifugation (100,000×*g*, 4 °C, 90 min) and resuspended in 1 × PBS. The particle sizes were evaluated by NTA. The experiments were repeated 3 times.

### Cell viability assay

Cell Counting Kit-8 (CCK-8, Dojindo Chemical Technology Co. Ltd, Shanghai, China) assays with Nthy-ori 3-1 and 8505C cells were carried out to evaluate the safety of iRGD-Exos in vitro [20, 28]. Briefly, cells were cultured in a 96-well plate at 5000/well in corresponding complete medium (100 μL) in an atmosphere of 5% CO_2_ at 37 °C for 24 h. Subsequently, the stale culture medium in each well was replaced with 100 μL of fresh complete medium containing different concentrations of iRGD-Exos (0 μg/mL, 50 μg/mL, 100 μg/mL, 200 μg/mL, 500 μg/mL, 1000 μg/mL). After an additional 24 h of culture, the medium in each well was replaced by 100 μL of CCK-8 working solution. Then, after another 2 h of incubation, the viabilities of the cells in each well were determined by measuring the absorbance at 450 nm with a microplate reader (BioTek, USA).

### In vitro cellular uptake study

A PKH26 Red Fluorescent Cell Linker Kit was purchased from Sigma (St. Louis, MO, USA) and used to label blank-Exos and iRGD-Exos as described by the manufacturer [28]. Briefly, purified blank-Exos were incubated with 0.4 μL of PKH26 and 200 μL of diluent C for 3 min, and then 200 μL of FBS was added to terminate staining. After washing twice with 1 × PBS and centrifugation (100,000×*g*, 90 min, 4 ℃), PKH26-iRGD-Exos were obtained and resuspended in 1 × PBS. PKH26-blank-Exos were also obtained using the same method.

8505C cells were seeded into 6-well plates at a density of 1 × 10^4^ cells/well and incubated overnight in an atmosphere of 5% CO_2_ at 37 °C. Cells were incubated with PKH26-blank-Exos (30 μg) or PKH26-iRGD-Exos (30 μg) for 4 h at 37 °C. Then, the cells were washed three times with 1 × PBS and fixed with paraformaldehyde (4% in 1 × PBS) for 30 min. Subsequently, the cells were stained with DAPI (1:1000 diluted with 1 × PBS) for 20 min. Finally, the cells were imaged by confocal microscopy (Zeiss, Jena, Germany).

The cellular uptake efficiency was further quantified with a flow cytometry assay [21, 34]. Briefly, 30 μg of PKH26-blank-Exos and PKH26-iRGD-Exos was added to 8505C cells and incubated for 3 and 6 h, respectively. Subsequently, the cells were collected, fixed with 4% paraformaldehyde, and analyzed by using a BD Biosciences flow cytometer (Franklin Lake, NJ, USA).

### Tumor-bearing nude mouse model

Four- to five-week-old female nude mice (BALB/c) were purchased from the Model Animal Center of Nanjing University and housed in a Tianjin Medical University specific pathogen-free (SPF) animal room. 8505C cells (1 × 10^7^ cells per mouse) were transplanted into the right hips of the mice. All animal experimental procedures were approved by the Institutional Animal Ethical and Welfare Committee of Tianjin Medical University.

### Tumor imaging, biodistribution and targeting in vivo

When the 8505C tumors grew to a diameter of approximately 10 mm, the mice were randomly divided into three groups to be used for tumor targeting validation and iRGD-Exos distribution determination of in vivo. The control, nontargeted and targeted groups were injected with PBS, DiR-labeled blank-Exos and DiR-labeled iRGD-Exos (1 mg/mL, 200 μL per mouse) via the tail vein, respectively. Then, at 0 h, 1 h, 8 h, and 24 h post-injection, images of the mice were captured using an IVIS Spectrum imaging system (Caliper Life Sciences, USA) [34].

In addition, SPECT/CT imaging was performed on each tumor-bearing mouse by tail vein injection of a relatively low-activity (7.4 MBq) imaging agent (Na^131^I, blank-Exos-^131^I or iRGD-Exos-^131^I). SPECT/CT images were captured 0 h, 0.5 h, 24 h, 72 h, and 96 h after the respective drug injection using a SPECT/CT scanner (GE Discovery NM/CT 670; GE Healthcare, Chicago, USA). During the image scanning period, the mice were maintained under anesthesia using 4% chloral hydrate (150 μL/mouse; Sigma-Aldrich, USA). Importantly, 7 days prior to intravenous compound injection, the mice began receiving NaI (1 mg/mL) in their drinking water to prevent exposure of the thyroid tissue to unwanted radiation during imaging.

### Pharmacokinetic study of Dox@iRGD-Exos-^131^I in vivo

For the pharmacokinetic study, 8505C tumor-bearing mice (n = 5) were administered DiR-labeled Dox@iRGD-Exos-^131^I (5 mg/mL, 200 μL) via tail vein injection. Then, at 0.5 h, 1 h, 2 h, 4 h, 8 h, 12 h, 24 h, and 48 h postinjection, 50 μL of orbital venous blood was collected from each mouse. The fluorescence intensity of each sample was measured with an IVIS fluorescence spectrometer (Caliper Life Sciences, USA).

### In vivo anti-tumor efficacy and biosafety

First, we established an ATC model by subcutaneous injection of 8505C cells into mice. When the tumors grew to a diameter of approximately 8 mm, the tumor-bearing mice were randomly divided into six groups (n = 30): PBS, iRGD-Exos, Na^131^I, blank-Exos-^131^I, iRGD-Exos-^131^I, and Dox@iRGD-Exos-^131^I. Then, each tumor-bearing mouse was injected with the corresponding drug combination via the tail vein (5 mg/mL, 200 μL; 5 mg/kg Dox; 74 MBq/mouse). To prevent thyroid tissue exposure to unwanted radiation, NaI (1 mg/mL) was added to the drinking water for all of mice 7 days before intravenous drug injection. Changes in body weight and tumor volume were measured every 3 days during the observation period. Tumor volumes were calculated using the following formula [20, 28]:

2 $$V=\frac{a{b}^{2}}{2}$$

where V is the tumor volume (mm^3^), a is the tumor length (mm), and b is the tumor width (mm). After the experiment, the tumor-bearing mice were sacrificed, venous blood was collected, the tumors and major solid organs (heart, liver, spleen, lung and kidney) were harvested, and the tumors were photographed and weighed. Venous blood was centrifuged at 3000×*g* for 8 min, and the serum levels of alanine transaminase (ALT) and creatinine (Cr) from the different treatment groups were measured to evaluate the biosafety of the as-developed multifunctional exosomes.

### Statistical analysis

All data are expressed as the mean ± SD. Statistical analysis was performed with IBM SPSS 26.0 software (IBM Corp, Armonk, NY, USA). A two-tailed Student’s t test was applied to determine the statistical significance of the differences between two groups, and one-way analysis of variance (ANOVA) was applied to examine the statistical significance of the differences among three or more groups. *P* < 0.05 was considered statistically significant. *P < 0.05, **P < 0.01, and ***P < 0.001.

## Results and discussion

### Expression of integrin αvβ3 in ATC

**Fig. 1:**
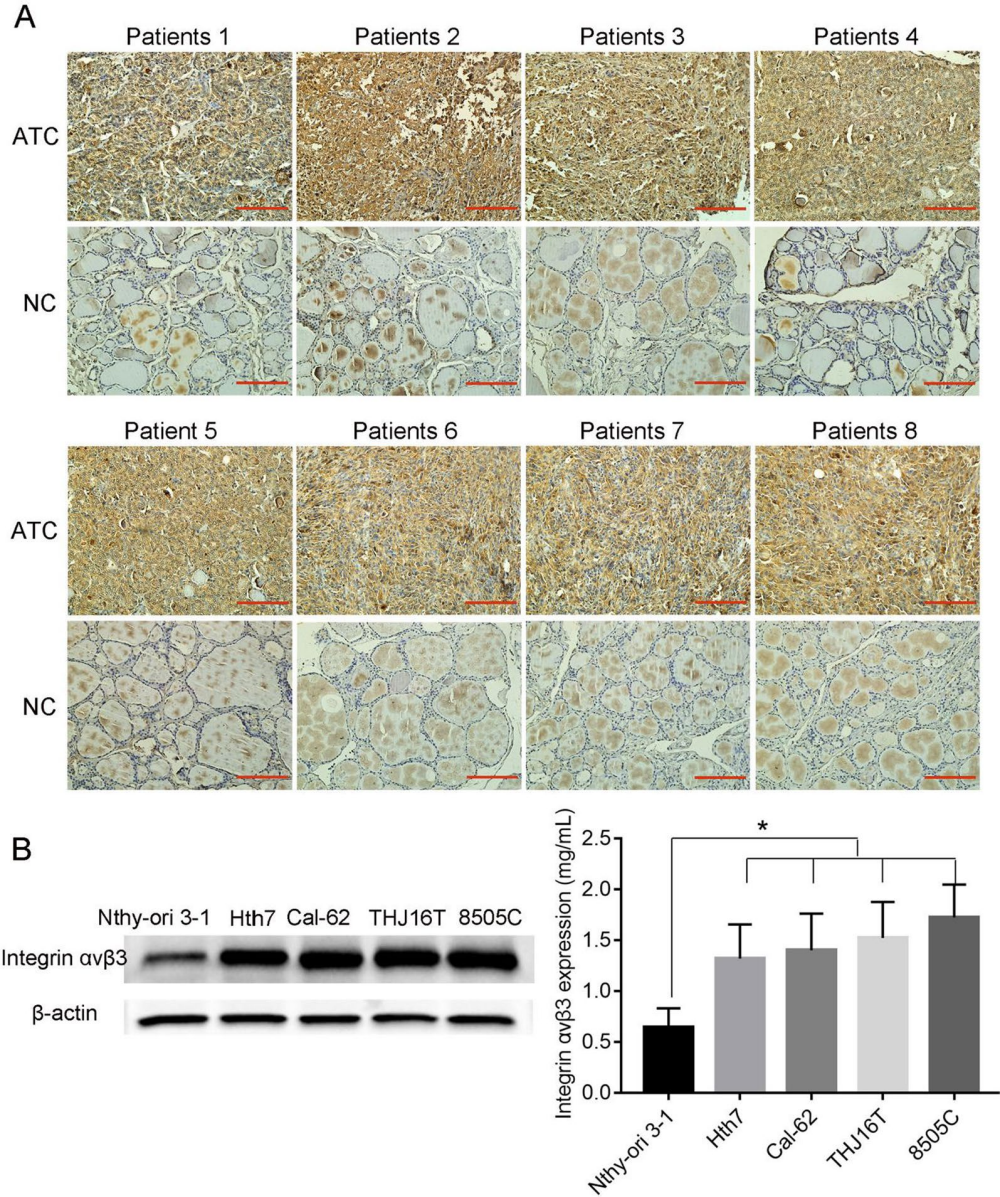
Expression level of integrin αvβ3 in ATC. **A** Immunohistochemistry of the paraffin-embedded human ATC cancerous tissues and paracancerous normal tissues to determine the expression of integrin αvβ3 (n = 8, 16 samples, scale bar = 10 μm). **B** Western blotting analysis of integrin αvβ3 expression in ATC cell lines (Hth7, Cal-62, THJ16T, and 8505C) and a normal thyroid cell line. Gray analysis was performed by ImageJ, *P < 0.05

Integrin αvβ3, an integrin family member, is a cell surface receptor that mediates cell adhesion and plays a crucial role in the occurrence, development and metastasis of solid tumors [30, 32]. Relevant studies have shown that integrin αvβ3 is highly expressed in a variety of malignant tumors (including melanoma [42], liver [43], and breast cancer [36]) and has become a keen target of interest in oncotherapy. ATC is a highly lethal form of thyroid carcinoma [44–46]. To assess the expression of integrin αvβ3 in ATC, 8 pairs of cancerous tissues and their adjacent noncancerous tissues from ATC patients who underwent surgery at Tianjin Medical University General Hospital were collected and evaluated by immunohistochemistry. Figure 1A shows that the expression of integrin αvβ3 in cancerous tissues was significantly higher than that in the adjacent noncancerous tissues.

In addition, the levels of integrin αvβ3 in ATC cells (Hth7, 8505C, THJ16T and Cal-62) and normal thyroid cells (Nthy-ori 3-1) were detected by western blot, and we found that the expression of integrin αvβ3 was significantly upregulated in ATC cells compared with that in Nthy-ori 3-1 cells (Fig. 1B). Thus, we regarded integrin αvβ3 as a potential therapeutic target for ATC.

### Construction and characterization of Dox@iRGD-Exos-^131^I

The tumor-penetrating peptide iRGD strictly binds to integrin αvβ3 and has been shown to increase the accumulation of drugs when conjugated to particle surfaces or codelivered [33, 47]. Based on this, we combined the exosomal membrane protein Lamp2b with iRGD peptide to improve the tumor targeting ability of our multifunctional exosomes.

**Fig. 2:**
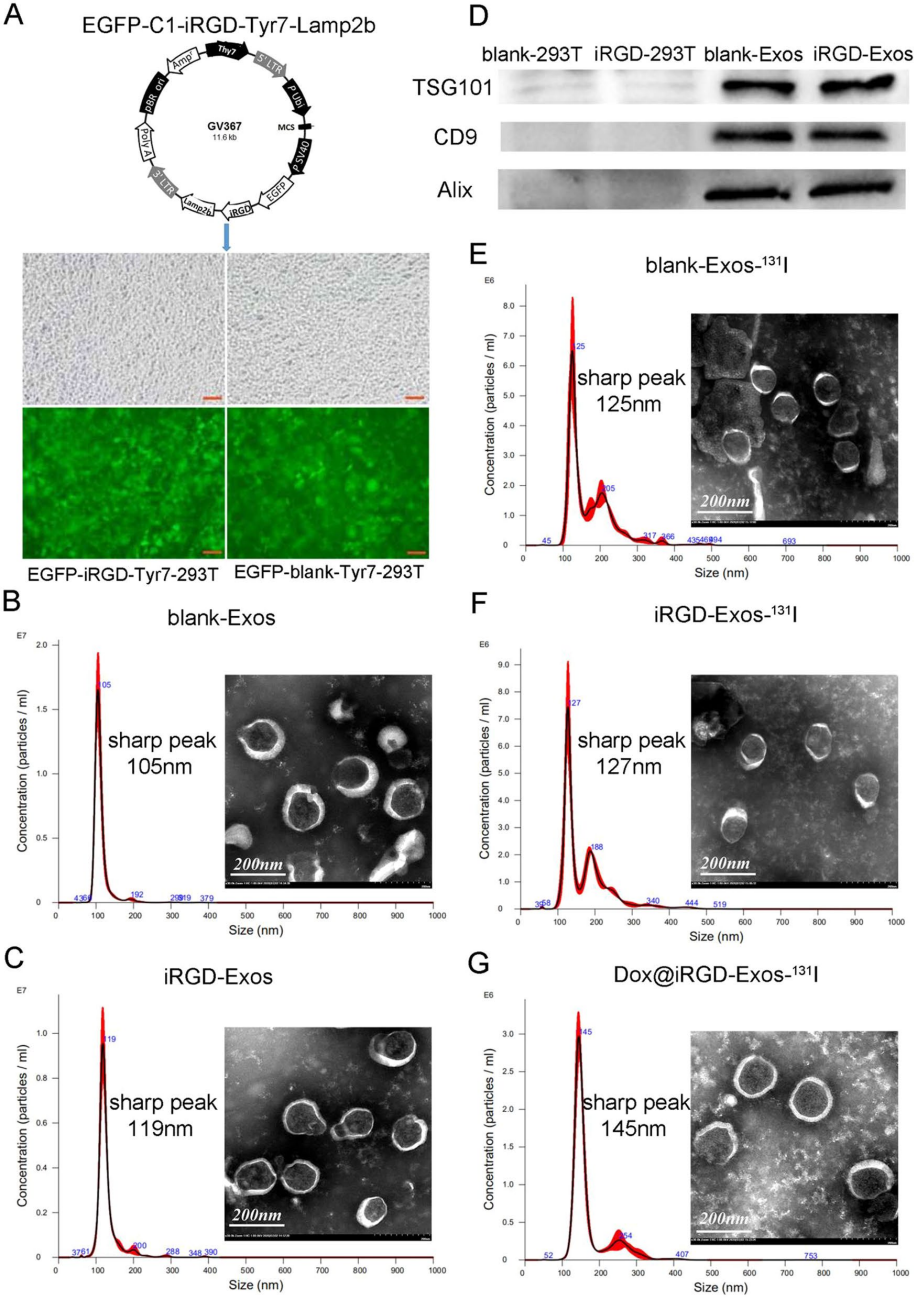
Characterization of Dox@iRGD-Exos-^131^I. **A** The main composition of the EGFP-C1-iRGD-Tyr7-Lamp2b plasmid and an image of iRGD/blank-Tyr7-EGFP-293T cells using fluorescence microscopy (scale bar = 100 μm). Representative TEM images and particle size distribution of **B** blank-Exos, **C** iRGD-Exos, **E** blank-Exos-^131^I, **F** iRGD-Exos-^131^I and **G** Dox@iRGD-Exos-^131^I (scale bar = 200 nm). **D** Western blotting analysis of exosome marker proteins (TSG101, CD9 and Alix) of blank-Exos and iRGD-Exos

To generate iRGD-overexpressed tyrosine exosomes (iRGD-Exos) and blank tyrosine exosomes (blank-Exos), we transfected HEK-293T cells with lentiviral vectors carrying EGFP-C1-iRGD-Tyr7-Lamp2b or the negative control EGFP-C1-blank-Tyr7-Lamp2b and verified successful transfection by fluorescence microscope (Fig. 2A). Then, iRGD-Exos and blank-Exos were separated from the cell culture supernatants by gradient differential centrifugation. As shown in Table 1, blank-Exos and iRGD-Exos showed average sizes of 112.1 ± 20.4 nm and 127.9 ± 26.4 nm; PDIs of 0.19 ± 0.05 and 0.27 ± 0.03; and zeta potentials of -38.86 ± 3.61 mV and -26.73 ± 3.12 mV, respectively. Furthermore, NTA measurements revealed that both blank-Exos and iRGD-Exos had a physically homogeneous particle size distribution, with sharp peaks at 105 nm and 119 nm, respectively (Fig. 2B, C), which is consistent with the size of typical exosomes reported previously [34, 36, 48, 49]. Additionally, the corresponding representative TEM images showed that blank-Exos and iRGD-Exos had spherical vesicle morphologies (Fig. 2B, C). Moreover, we verified the expression of relevant exosome marker proteins (Alix, TSG101 and CD9) in blank-Exos and iRGD-Exos by western blot, which indicated that the vesicles were 293T-derived exosomes (Fig. 2D).

**Table 1:** Summary and comparison of the mean sizes, polydispersity indices, and zeta potentials of various samples

Sample	Size (nm)	Polydispersity index (PDI)	Zeta potential (mV)
blank-Exos	112.1 ± 20.4	0.19 ± 0.05	− 38.86 ± 3.61
iRGD-Exos	127.9 ± 26.4	0.27 ± 0.03	− 26.73 ± 3.12
blank-Exos-^131^I	164.4 ± 61.2	0.51 ± 0.06	− 24.03 ± 1.91
iRGD-Exos-^131^I	172.8 ± 65.8	0.57 ± 0.02	− 18.44 ± 5.43
Dox@iRGD-Exos-^131^I	170.1 ± 45.6	0.41 ± 0.05	− 9.03 ± 2.73

Subsequently, to fully use the tyrosine residues and the natural lipid bilayer of the engineered exosomes, we loaded Dox into the iRGD-Exos and labeled them with ^131^I. The TEM images showed that the morphologies of the exosomes remained as spherical vesicles, indicating that the exosome membrane structure was not damaged after loading with Dox or labeling with ^131^I by the chloramine-T method (Fig. 2E–G). In addition, compared with blank-Exos and iRGD-Exos, the hydrodynamic diameters of blank-Exos-^131^I, iRGD-Exos-^131^I and Dox@iRGD-Exos-^131^I increased according to DLS analysis, and their surface zeta potentials changed as well, indicating that Dox and ^131^I were successfully integrated into the corresponding exosomes (Table 1). We also observed that when labeled with ^131^I and/or loaded with Dox, the PDIs increased compared with those of blank-Exos and iRGD-Exos, which may be related to the aggregation of the exosomes during the process of loading Dox and labeling with ^131^I.

### Analysis of Dox loading into exosomes

The natural lipid bilayer structure and large surface area make exosomes particularly suitable for drug delivery [50–52]. Dox, a typical chemotherapy agent [13, 53, 54], was used to study the drug-loading efficiency of iRGD-Exos in this study, The UV–Vis spectrum of Dox@iRGD-Exos showed a clear absorption peak from Dox at λ = 485 nm [20, 55, 56] compared to that of iRGD-Exos, suggesting the successful integration of Dox into iRGD-Exos (Additional file 1: Fig. S1A). Furthermore, we measured the loading efficiency of Dox into iRGD-Exos according to the measured absorbance of Dox at λ = 485 nm (Additional file 1: Fig. S1B), and found by quantitative analysis that the Dox loading efficiency was 11.73%.

### ^131^I labeling and stability of the as-prepared exosomes

In this study, the ^131^I labeling efficiency was approximately 50.16%-60.21% (Additional file 1: Fig. S2A). The radiochemical purity was approximately 97.89%-100% after purification by three centrifugation steps (Additional file 1: Fig. S2B). As noted above, the as-developed Dox@iRGD-Exos were successfully labeled with ^131^I.

To verify the stability of the as-prepared Dox@iRGD-Exos-^131^I, we suspended blank-Exos and Dox@iRGD-Exos-^131^I in 1 × PBS at 4 °C and serum at 37 °C, respectively. The results showed that the sizes of blank-Exos and Dox@iRGD-Exos-^131^I (Additional file 1: Fig. S3A, B) did not change significantly over 7 days, indicating that the blank-Exos and the as-prepared exosomes can remain stable for a certain period of time and that iRGD modification, Dox loading and ^131^I labeling had no significant impact on the stability of the exosomes.

### In vitro targeting of iRGD-Exos

**Fig. 3:**
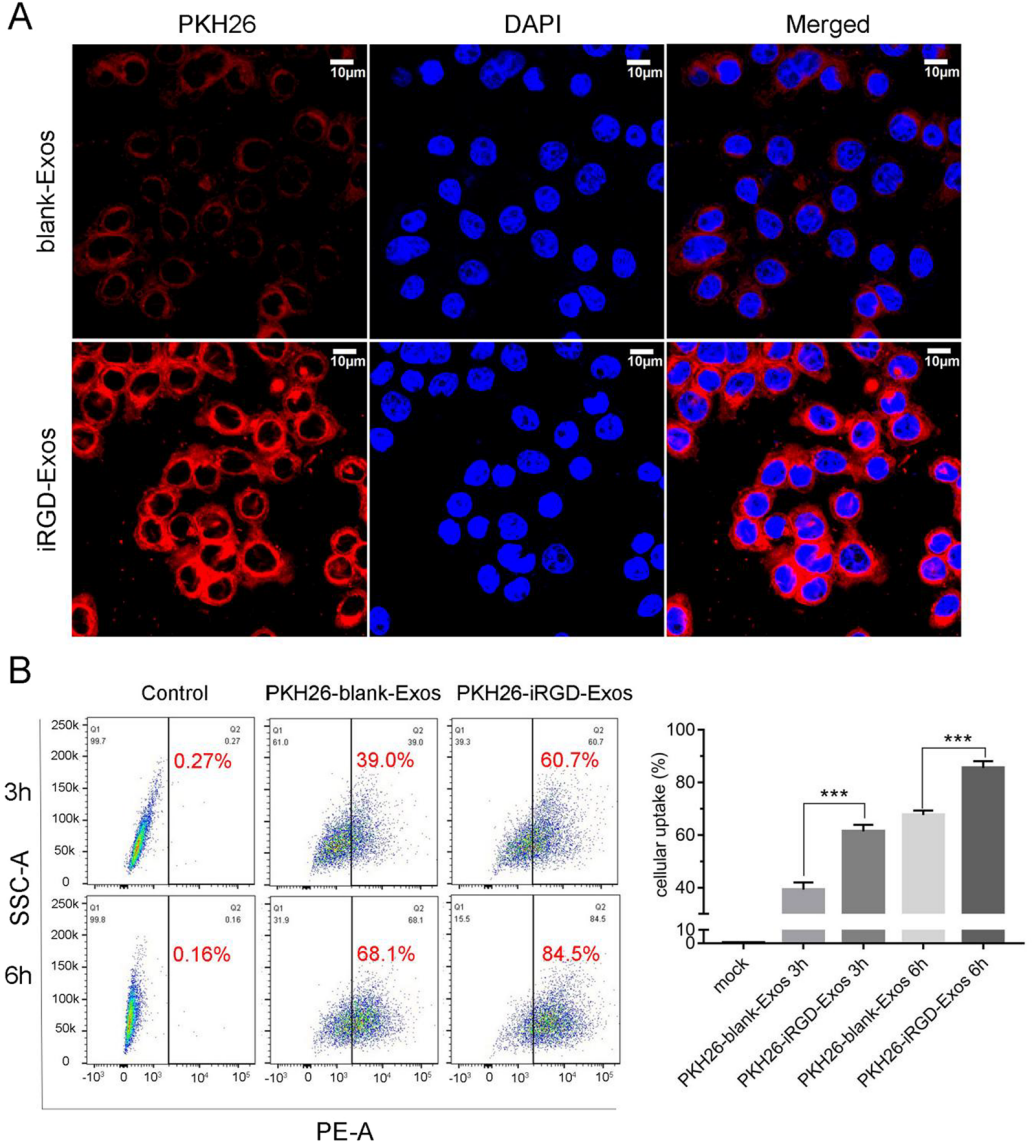
In vitro targeting of iRGD-Exos. **A** Confocal microscopy images of 8505C cells incubated with PKH26-blank-Exos and PKH26-iRGD-Exos at 4 h. Nuclei were stained with DAPI (blue). Fluorescence from PKH26 (red) and DAPI (blue) was observed. The scale bar is 10 μm. **B** Flow cytometric analysis of PKH26-iRGD-Exos binding to 8505C cells. Exosomes were labeled with PKH26 and incubated with 8505C for different lengths of time (3 h, or 6 h). ***P < 0.001

To evaluate the tumor-targeting ability of iRGD-Exos in vitro and investigate whether the iRGD peptide modification can enhance the binding capability of the HEK-293T exosomes to ATC cells, blank-Exos and iRGD-Exos were first labeled with PKH26 and cultured with 8505C cells. After incubation with 8505C cells for 4 h, two kinds of exosomes (blank-Exos and iRGD-Exos) were successfully phagocytized into the recipient cells, and a significantly higher PKH26 fluorescence signal was observed in the iRGD-Exos group (Fig. 3A). Additionally, to estimate cellular uptake efficiency, PKH26-blank-Exos and PKH26-iRGD-Exos were incubated with 8505C cells for 3 h or 6 h at 37 ℃, respectively. As shown in Fig. 3B, the iRGD-Exos group displayed higher cellular uptake than the blank-Exos group as analyzed by flow cytometry at the same incubation time, which was consistent with the in vitro results observed by the confocal microscopy. These results indicated that the iRGD peptide modification could significantly enhance the binding ability of the exosomes to 8505C cells.

### In vitro antitumor effect of Dox@iRGD-Exos-^131^I

**Fig. 4:**
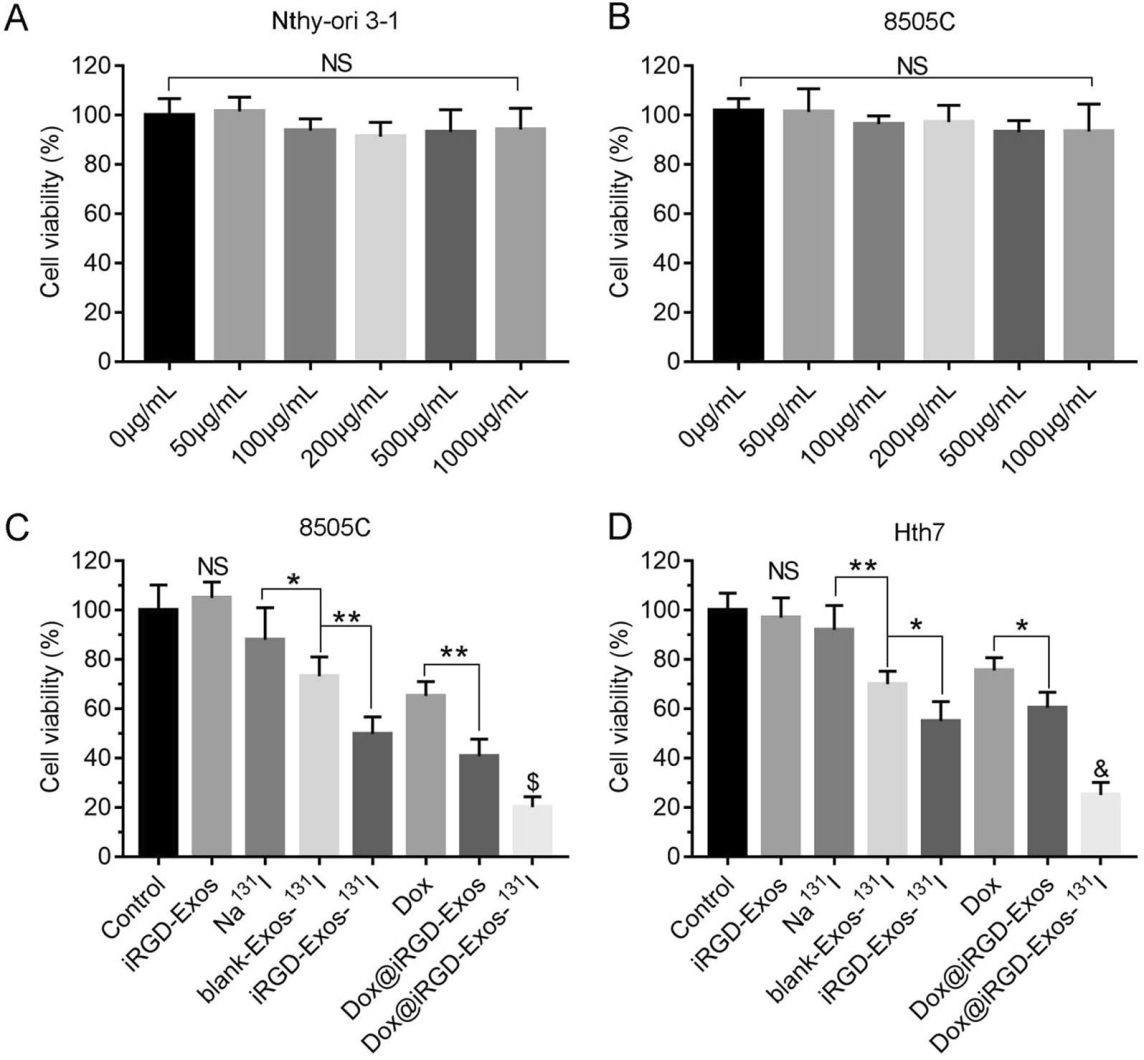
Cell viability assay. Viability of **A** Nthy-ori 3-1 and **B** 8505C cells treated with different concentrations of iRGD-Exos (0–1000 μg/mL) for 24 h. **C** 8505C and **D** Hth7 cells were incubated with control medium, iRGD-Exos, Na^131^I (3.7 MBq/well), blank-Exos-^131^I, iRGD-Exos-^131^I, Dox, Dox@iRGD-Exos, or Dox@iRGD-Exos-^131^I (15 μg/mL Dox) at 24 h at the same dose of radioactivity (3.7 MBq/well). A CCK-8 assay was used to assess cell viability in each group. NS (not significant) indicates *P* > 0.05 compared to the control group; *P < 0.05; **P < 0.01; $ indicates *P* < 0.01 compared to the other groups; & indicates *P* < 0.001 compared to the other groups

Before evaluating the therapeutic effect of Dox@iRGD-Exos-^131^I, the in vitro viabilities of Nthy-ori 3-1 and 8505C cells treated with various concentrations of iRGD-Exos (0–1000 μg/mL) were studied with a standard CCK-8 assay [20, 36]. As shown in Fig. 4A, B, cell viabilities were all above 95% even at the highest concentration of iRGD-Exos (1000 μg/mL), which confirmed that iRGD-Exos had excellent biocompatibility and were hardly toxic to normal cells.

Next, we analyzed the ability of Dox@iRGD-Exos-^131^I to inhibit 8505C cells. 8505C cells were treated with control medium, iRGD-Exos, free Na^131^I, blank-Exos-^131^I, iRGD-Exos-^131^I, Dox, Dox@iRGD-Exos, and Dox@iRGD-Exos-^131^I for 24 h. As shown in Fig. 4C, we found that the Na^131^I group showed limited cytotoxicity (cell viability above 80%), while blank-Exos-^131^I and iRGD-Exos-^131^I showed significantly stronger cytotoxicity than Na^131^I at the same radioactivity (3.7 MBq/well). Dox@iRGD-Exos inhibited cell growth significantly more strongly than free Dox (Additional file 1: Fig. S4; Fig. 4C), while no significant inhibition of cell proliferation was observed after treatment with iRGD-Exos, indicating that the exosomes themselves are nontoxic that Dox@iRGD-Exos can efficiently deliver chemotherapeutic drugs into tumor cells. Additionally, compared with the other groups, the Dox@iRGD-Exos-^131^I group showed the strongest inhibition of cell proliferation. Similar results were obtained for Hth7 cells (Fig. 4D), confirming that the as-developed Dox@iRGD-Exos-^131^I had a exhibited significant tumor inhibition in vitro.

### Pharmacokinetics of Dox@iRGD-Exos-^131^I in vivo

The pharmacokinetics of Dox@iRGD-Exos-^131^I were next investigated. As shown in Additional file 1: Fig. S5, blood circulation was calculated using a two-compartment blood circulation model, and the half-life was 7.81 h. This excellent blood retention and half-life make Dox@iRGD-Exos-^131^I more favorable for tumor accumulation.

### In vivo tumor targeting and biodistribution of iRGD-Exos

**Fig. 5:**
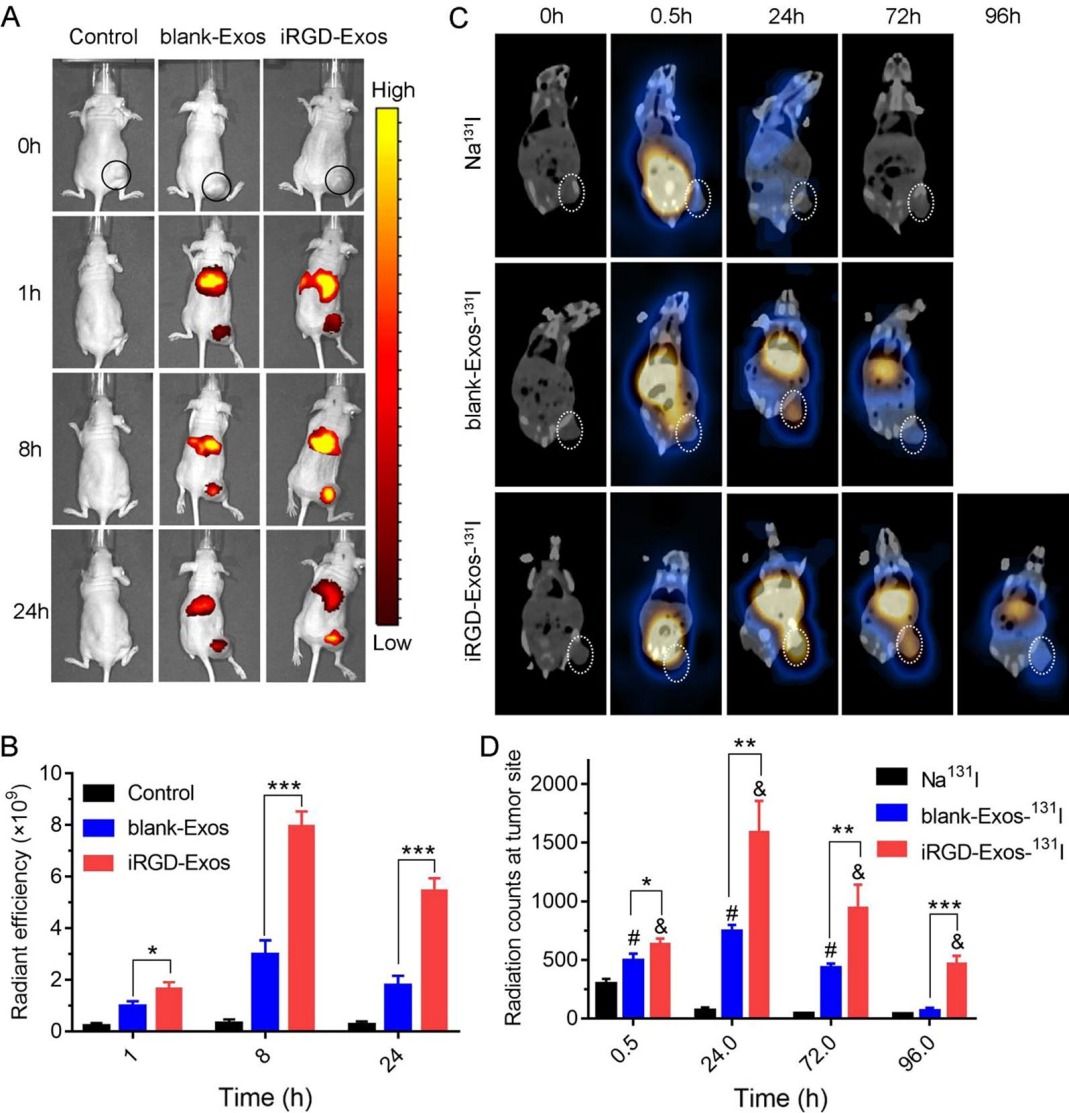
**A** In vivo fluorescence imaging of 8505C tumor-bearing nude mice at 0 h, 1 h, 8 h and 24 h after tail vein administration of DiR-labeled blank-Exos/iRGD-Exos. Black circle indicates the tumor site. **B** Quantitative analysis of the tumor fluorescence intensity of the in vivo images. **C** In vivo SPECT/CT imaging of 8505C tumor-bearing nude mice after intravenous injection of different drug combinations (Na^131^I, blank-Exos-^131^I, and iRGD-Exos-^131^I) at 0.5 h, 24 h, 72 h and 96 h postinjection. White circle indicates the tumor site.** D** Quantitative analysis of the radiation counts at the tumor site. *P < 0.05, **P < 0.01, ***P < 0.001. # indicates *P* < 0.01 compared to the Na^131^I group. & indicates *P* < 0.001 compared to the Na^131^I group

To evaluate the tumor-targeting efficency of iRGD-Exos in vivo, 8505C tumor-bearing mice were administered DiR-labeled blank-Exos/iRGD-Exos for in vivo imaging after tail vein injection. The biodistribution of blank-Exos/iRGD-Exos was observed at 0 h, 1 h, 8 h, and 24 h using an IVIS fluorescence imaging system. As shown in Fig. 5A, clear fluorescence signals were observed at the tumor site and in the liver in both groups at 1 h. Quantitative analysis of the fluorescence images indicated that iRGD-Exos exhibited higher accumulation in the tumor than blank-Exos at all predetermined time points (Fig. 5B), indicating that iRGD modification effectively enhanced the tumor targeting ability of these nanoparticles [34, 36].

To further observe the tumor-targeted distribution of ^131^I-labeled targeted/nontargeted exosomes, we labeled blank-Exos/iRGD-Exos with ^131^I and for tail vein injection 8505C tumor-bearing mice and in vivo imaging. The distribution of the exosomes was monitored at predetermined time points (0 h, 0.5 h, 24 h, 72 h and 96 h) using single-photon emission computed tomography-computed tomography (SPECT/CT). As shown in Fig. 5C, D, radioactive signals were observed at the tumor site in all three groups at 0.5 h postinjection. In addition, the radioactivity in the Na^131^I group cleared the fastest, and no radioactivity was observed in the tumor region at 24 h postinjection. In contrast, the radiation signal was most notable at 24 h post-injection in both the blank-Exos-^131^I and iRGD-Exos-^131^I groups. Additionally, the radioactivity at the tumor site was significantly higher in the iRGD-Exos-^131^I group than in the Na^131^I group and blank-Exos-^131^I group at each time point. Additionally, in the iRGD-targeted group, radioactivity was still be detected at the tumor site at the last timepoint (92 h post-injection) (Fig. 5D). These results indicated that iRGD-targeted exosomes could not only be more concentrated at ATC tumors, but also remain in tumor tissues for a longer time than nontargeted exosomes, providing evidence for subsequent ATC treatment.

### In vivo dual antitumor efficacy and biosafety

**Fig. 6:**
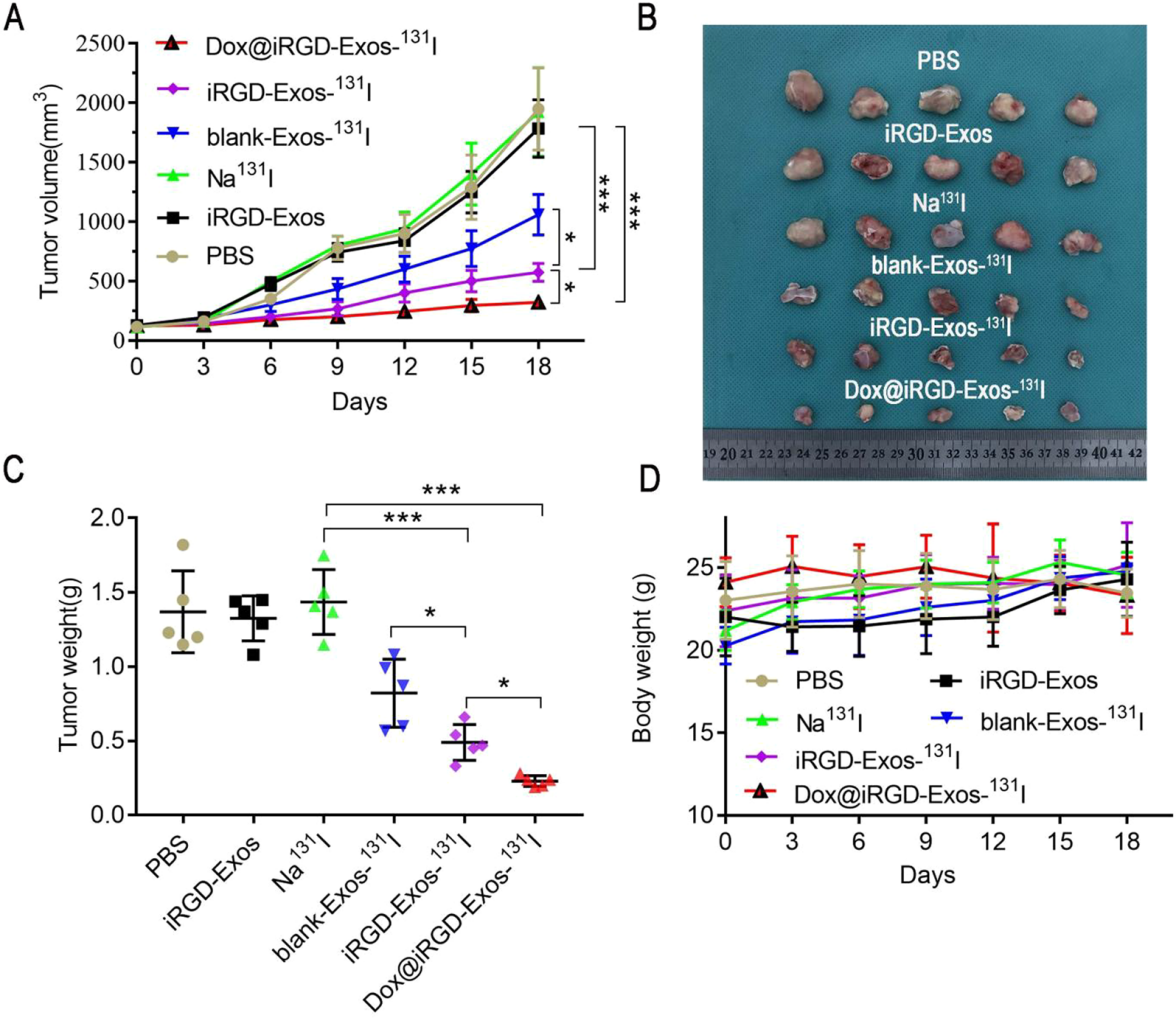
Antitumor efficacy in vivo. **A** Growth curves of the 8505C tumors after the mice were injected with PBS, iRGD-Exos, Na^131^I, blank-Exos-^131^I, iRGD-Exos-^131^I or Dox@iRGD-Exos-^131^I. Tumor volume was measured every 3 days until the end of the observation period. **B** Ex vivo image of the tumors from the sacrificed mice at 18 days postinjection. **C** Quantitative analysis of the tumor weights from the mice in the different treatment groups. **D** Changes in body weights of the different drug-treated mice during the observation period. Data are presented as the mean ± SD. *P < 0.05, ***P < 0.001

Encouraged by the fascinating tumor accumulation in vivo and excellent inhibitory ability of Dox@iRGD-Exos-^131^I in 8505C cells, we investigated the therapeutic effect of the different treatments and the potential dual antitumor effects of Dox@iRGD-Exos-^131^I on tumor-bearing mice using an 8505C xenograft mouse model. When the tumors reached a diameter of approximately 8 mm, the 8505C tumor-bearing mice were intravenously injected with different drug combinations. As shown in Fig. 6A, tumor volume increased significantly over time in the PBS, iRGD-Exos and Na^131^I groups. However, tumor volume growth gradually slowed to varying degrees in the other 3 groups, with the Dox@iRGD-Exos-^131^I group showing the slowest growth; these tumors had shrunk to six times smaller than the tumors in the PBS group at the end of observation. Additionally, the tumor tissue was removed from each mouse at the end of observation. The ex vivo tumor images (Fig. 6B) and tumor weights from each group (Fig. 6C) visually illustrate the therapeutic effects, with Dox@iRGD-Exos-^131^I displaying the strongest inhibitory effect on tumor growth. This excellent antitumor effect was attributed to not only the dual codelivery effects of Dox and ^131^I on tumors, but also the enhanced targeting function of the modified iRGD peptides. Of course, their critical link is the exosome as the codelivery vehicle. Importantly, no notable loss in body weight was observed in any of the six groups (Fig. 6D).

To assess the biosafety of the multifunctional exosomes, at the end of the observation period, mouse serum was collected to measure the levels of ALT and Cr, which are commonly used as biomarkers of liver and kidney injury, respectively. As shown in Additional file 1: Fig. S6A, the level of ALT in the iRGD-Exos group was not significantly different from that in the PBS group, indicating that iRGD-Exos were nontoxic. More importantly, the level of ALT was significantly lower in the Dox@iRGD-Exos-^131^I group than in the blank-Exos-^131^I group and similar to that in iRGD-Exos-^131^I-treated mice, indicating that Dox@iRGD-Exos-^131^I is less hepatotoxic than blank-Exos-^131^I. Additionally, the levels of Cr in all treatment groups were not significantly different (Additional file 1: Fig. S6B), indicating that Dox@iRGD-Exos-^131^I may not cause obvious damage to the kidney.

**Fig. 7:**
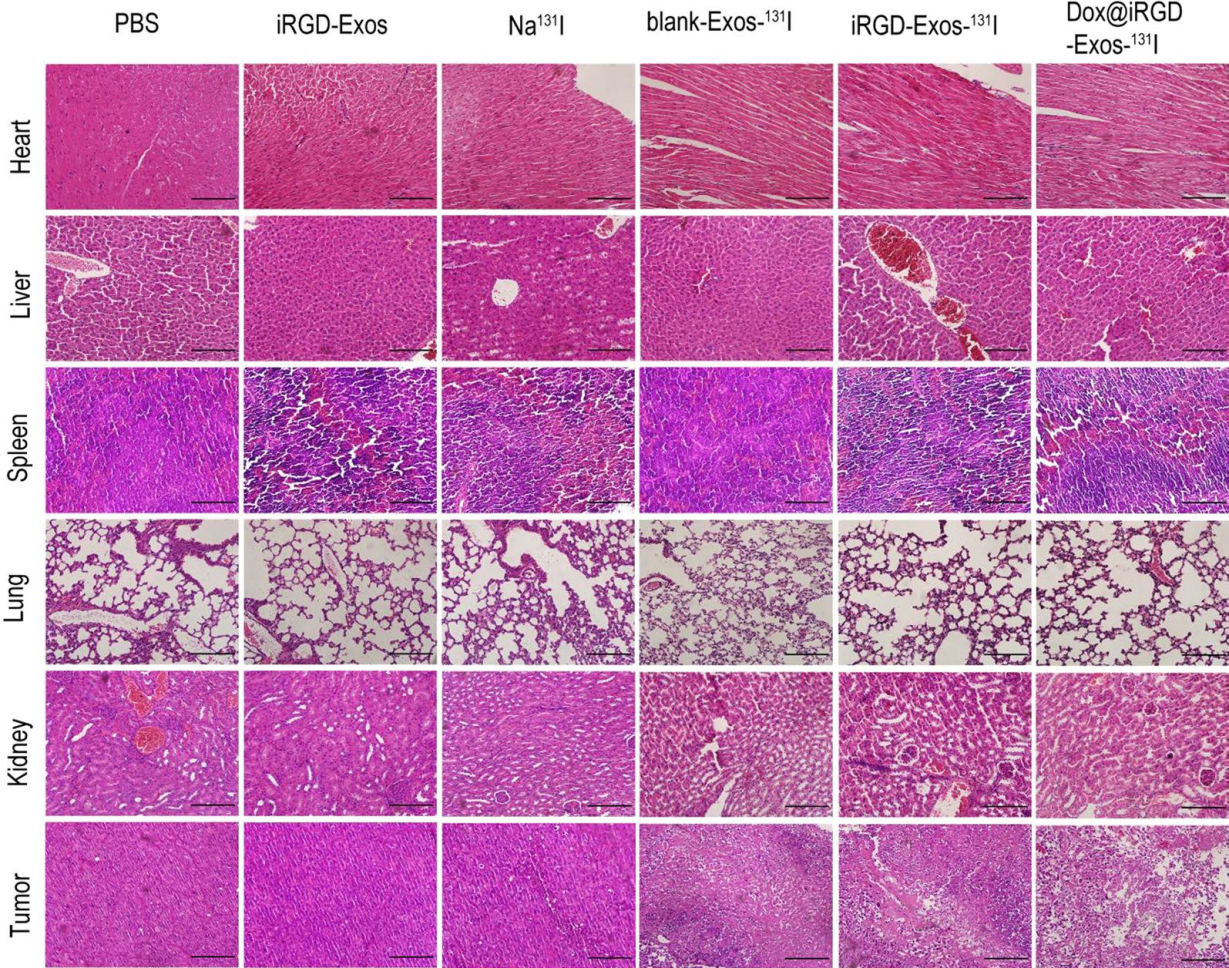
Representative H&E-stained images of the tumors and major organs (heart, liver, spleen, lung, and kidney) collected from 8505C tumor-bearing mice in different treatment groups after sacrifice. The scale bar represents 10 μm

At the end of the observation period, we examined tumor apoptosis and histological changes in the major organs (heart, liver, spleen, lung, and kidney) induced by the different drug treatments using a hematoxylin and eosin (H&E) staining assay. As shown in Fig. 7, no significant changes were observed in the H&E staining images of the major organs in the 5 experimental groups compared with the PBS group, indicating no obvious side effects and good biosafety of each treatment, including Dox@iRGD-Exos-^131^I. Additionally, no significant pathological changes or inflammation were observed in tumor tissues collected from the PBS-, iRGD-Exos- and Na^131^I-treated groups. However, different degrees of tumor tissues necrosis was observed in the remaining 3 groups, with the most serious necrosis found in the Dox@iRGD-Exos-^131^I-treated group. The in vivo results further highlighted the dual advantages of chemotherapy combined with internal irradiation therapy based on the developed engineered exosomes, as Dox@iRGD-Exos-^131^I exhibits several favorable and provides a novel and promising therapeutic strategy for ATC.

## Conclusions

In summary, we developed a novel dual antitumor strategy combining internal irradiation and chemotherapy by means of iRGD-targeted exosomes as a delivery vector, which was capable of codelivering ^131^I and Dox to ATC cells efficiently and precisely. The iRGD-targeted exosomes were more concentrated in the ATC cells or tumors in vivo and in vitro, confirming the enhanced targeting function of iRGD peptide modification. After intravenous injection of Dox@iRGD-Exos-^131^I, 8505C tumor-bearing mice exhibited significant tumor inhibition with no obvious side effects. To our knowledge, this is the first report of the application of the engineered exosomes for ATC treatment using therapeutic radionuclides and chemotherapy for dual functions. These as-developed multifunctional exosomes have excellent tumor targeting ability and dual therapeutic effects, providing novel insight into current ATC treatment and holding great potential for improving therapeutic efficacy against a wide range of integrin αvβ3-overexpressing tumors.

## Supplementary Information

**Additional file 1: Fig. S1.** (A) UV–Vis spectra of the Dox, iRGD-Exos, and Dox@iRGD-Exos from 250 to 700 nm. (B) Standard concentration curve of Dox constructed with measurements made by a UV-3600 plus UV–Vis spectrophotometer. **Fig. S2.** Radiochemical efficiency and purity of ^131^I-labeled Dox@iRGD-Exos. (A) Radiochemical efficiency and (B) purity were measured by instant thin-layer chromatography (TLC) with an AR-2000 radio-TLC imaging scanner. **Fig. S3.** Stability of the as-prepared exosomes. The changes in diameter of (A) blank-Exos and (B) Dox@iRGD-Exos-^131^I at 4 °C in 1 × PBS and at 37 °C in serum, respectively, over 7 days. Data are shown as the mean ± SD (n = 3). **Fig. S4.** Cell viability assay. Viability of (A) 8505C and (B) Hth7 cells treated with different concentrations of Dox or Dox@iRGD-Exos for 24 h. A CCK-8 assay was used to assess cell viability in each group. NS, not significant; * indicates *P* < 0.05 compared to the Dox group at the same concentration; ** indicates *P* < 0.01 compared to the Dox group at the same concentration. **Fig. S5.** Blood circulation half-life of Dox@iRGD-Exos-^131^I in 8505C tumor-bearing mice after intravenous injection. Data represent the mean ± SD (n = 5). **Fig. S6.** Biosafety assessment of the multifunctional exosomes. The levels of (A) ALT and (B) Cr in serum collected from the mice in the different treatment groups. Alanine transaminase, ALT; creatinine, Cr; not significant, NS; *P < 0.05; **P < 0.01; ***P < 0.001.

## Data Availability

The datasets used during the present study are available from the corresponding author upon reasonable request.
