# Supplementary material for: Engineering a HEK-293T exosome-based delivery platform for efficient tumor-targeting chemotherapy/internal irradiation combination therapy
Source: J Nanobiotechnology. 2022 May 31;20:247. doi: 10.1186/s12951-022-01462-1 (PMC9153154; doi:10.1186/s12951-022-01462-1)
Supplement: Supplementary file 1 — Additional file 1: Fig. S1. (A) UV–Vis spectra of the Dox, iRGD-Exos, and Dox@iRGD-Exos from 250 to 700 nm. (B) Standard concentration curve of Dox constructed with measurements made by a UV-3600 plus UV–Vis spectrophotometer. Fig. S2. Radiochemical efficiency and purity of 131I-labeled Dox@iRGD-Exos. (A) Radiochemical efficiency and (B) purity were measured by instant thin-layer chromatography (TLC) with an AR-2000 radio-TLC imaging scanner. Fig. S3. Stability of the as-prepared exosomes. The changes in diameter of (A) blank-Exos and (B) Dox@iRGD-Exos-131I at 4 °C in 1 × PBS and at 37 °C in serum, respectively, over 7 days. Data are shown as the mean ± SD (n = 3). Fig. S4. Cell viability assay. Viability of (A) 8505C and (B) Hth7 cells treated with different concentrations of Dox or Dox@iRGD-Exos for 24 h. A CCK-8 assay was used to assess cell viability in each group. NS, not significant; * indicates P < 0.05 compared to the Dox group at the same concentration; ** indicates P < 0.01 compared to the Dox group at the same concentration. Fig. S5. Blood circulation half-life of Dox@iRGD-Exos-131I in 8505C tumor-bearing mice after intravenous injection. Data represent the mean ± SD (n = 5). Fig. S6. Biosafety assessment of the multifunctional exosomes. The levels of (A) ALT and (B) Cr in serum collected from the mice in the different treatment groups. Alanine transaminase, ALT; creatinine, Cr; not significant, NS; *P < 0.05; **P < 0.01; ***P < 0.001. [file 12951_2022_1462_MOESM1_ESM.docx]

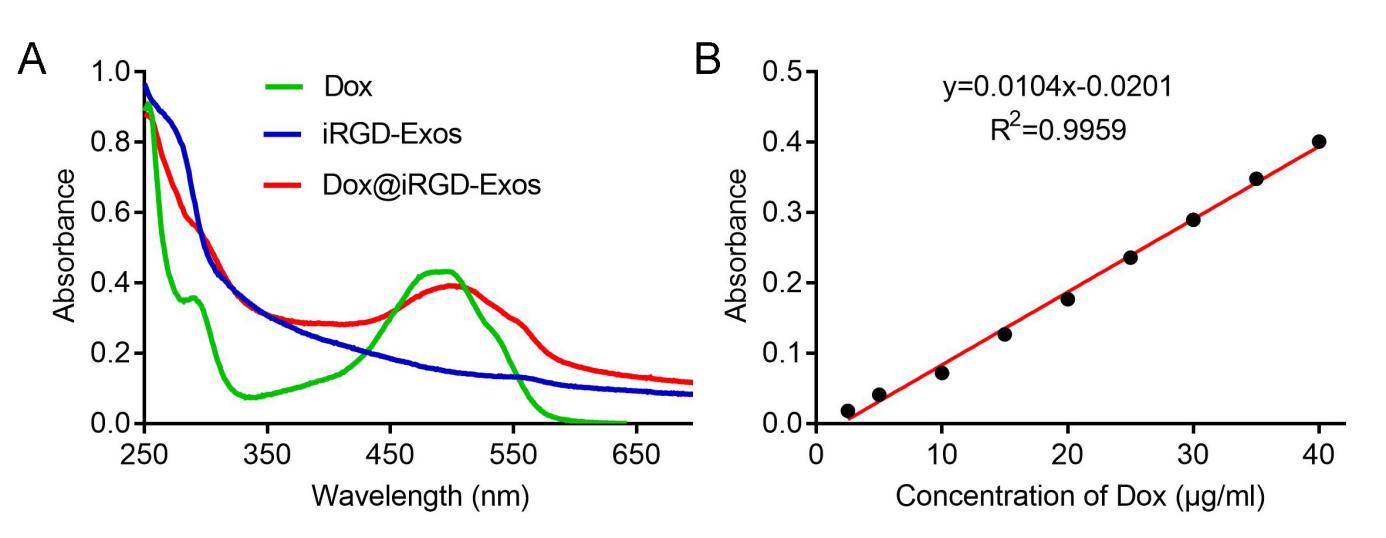


***Legend***

**Fig. S1:** (A) UV-Vis spectra of the Dox, iRGD-Exos, and Dox@iRGD-Exos from 250 to 700 nm. (B) Standard concentration curve of Dox constructed with measurements made by a UV-3600 plus UV-Vis spectrophotometer.


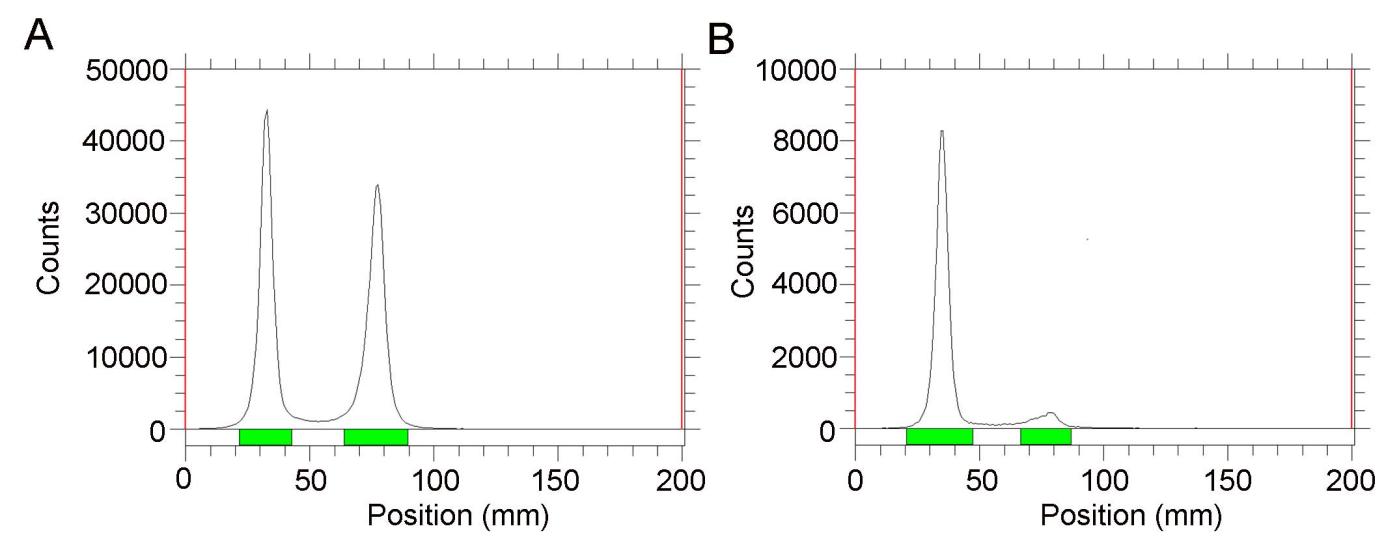


***Legend***

**Fig. S2:** Radiochemical efficiency and purity of ^131^I-labeled Dox@iRGD-Exos. (A) Radiochemical efficiency and (B) purity were measured by instant thin-layer chromatography (TLC) with an AR-2000 radio-TLC imaging scanner.

***
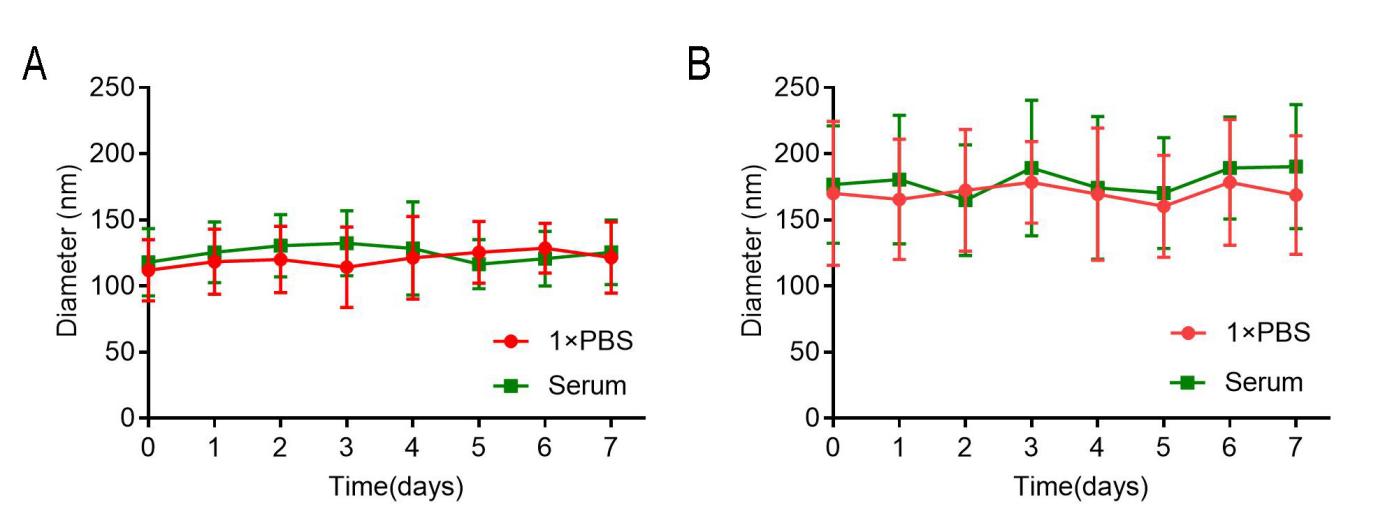
***

***Legend***

**Fig. S3:** Stability of the as-prepared exosomes. The changes in diameter of (A) blank-Exos and (B) Dox@iRGD-Exos-^131^I at 4 °C in 1×PBS and at 37 °C in serum, respectively, over 7 days. Data are shown as the mean ± SD (n=3).

***
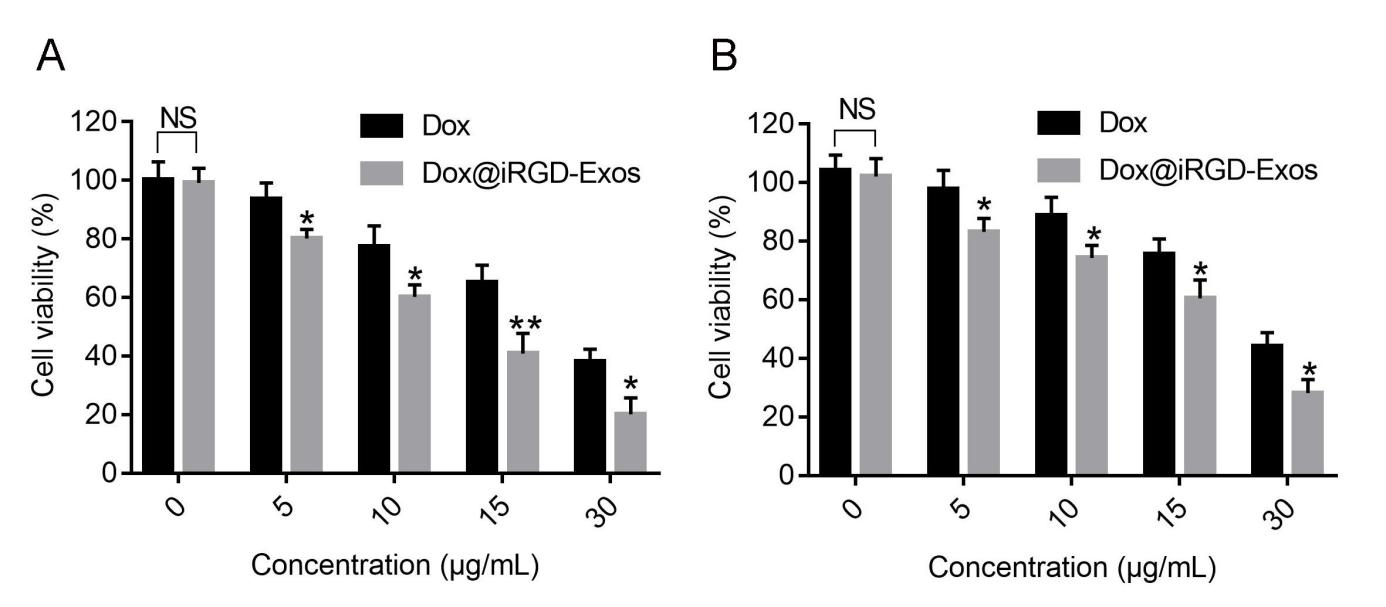
***

***Legend***

**Fig. S4:** Cell viability assay. Viability of (A) 8505C and (B) Hth7 cells treated with different concentrations of Dox or Dox@iRGD-Exos for 24 h. A CCK-8 assay was used to assess cell viability in each group. NS, not significant; * indicates *P* < 0.05 compared to the Dox group at the same concentration; ** indicates *P* < 0.01 compared to the Dox group at the same concentration.


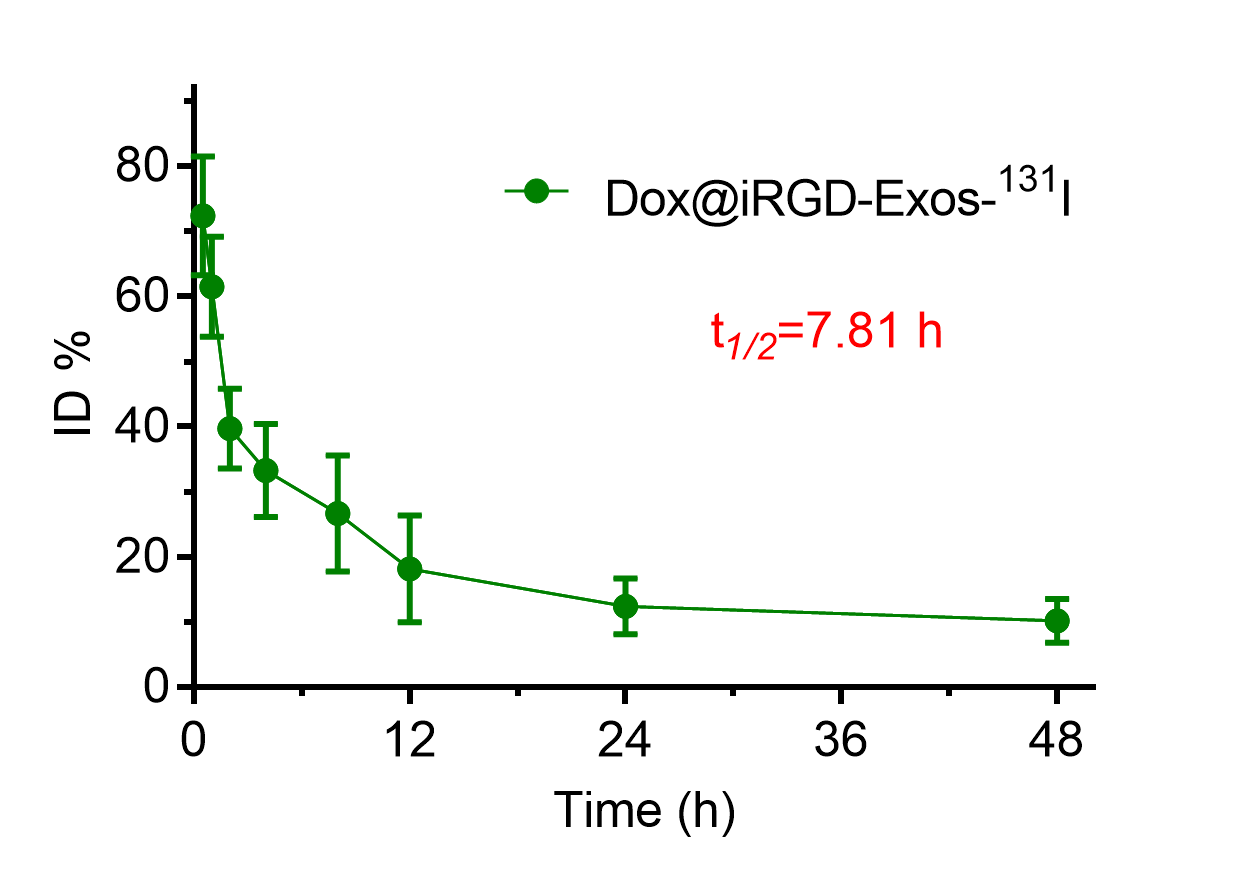


***Legend***

**Fig. S5:** Blood circulation half-life of Dox@iRGD-Exos-^131^I in 8505C tumor-bearing mice after intravenous injection. Data represent the mean ± SD (n=5).


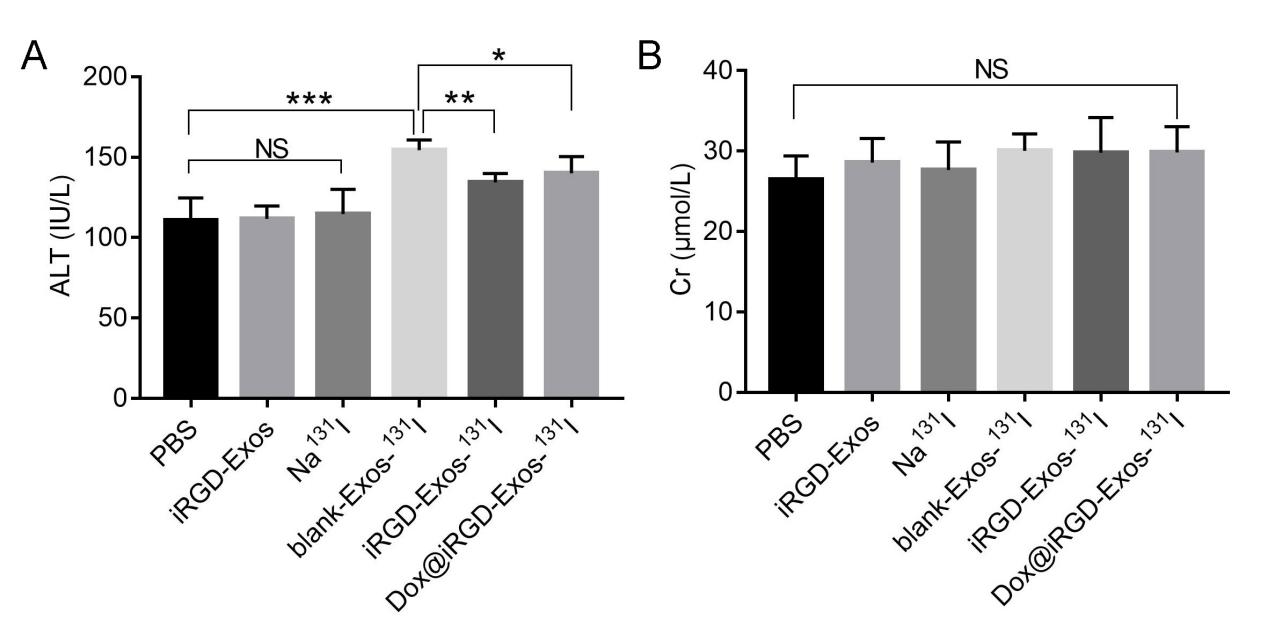


***Legend***

**Fig. S6:** Biosafety assessment of the multifunctional exosomes. The levels of (A) ALT and (B) Cr in serum collected from the mice in the different treatment groups. Alanine transaminase, ALT; creatinine, Cr; not significant, NS; **P* < 0.05; ***P* < 0.01; ****P* < 0.001.
